# Cervical muscle stiffness and parasympathetic nervous system improvements for treatment-resistant depression

**DOI:** 10.1186/s12891-022-05860-2

**Published:** 2022-10-11

**Authors:** Takayoshi Matsui, Kazuhiro Hara, Makoto Iwata, Shuntaro Hojo, Nobuyuki Shitara, Yuzo Endo, Hideoki Fukuoka, Masaki Matsui, Hiroshi Kawaguchi

**Affiliations:** 1 Tokyo Neurological Center, Toranomon 4-1-17, Minato-ku, Tokyo, 105-0001 Japan; 2 Matsui Hospital, Kan-nonji 739, Tokyo, Kagawa 768-0013 Japan

**Keywords:** Treatment-resistant depression (TRD), Physical therapy, Cervical muscle, Parasympathetic nervous system

## Abstract

**Background:**

Although treatment-resistant depression (TRD) is a major public health problem that increases mortality due to suicides, a considerable percentage of patients do not respond adequately to variable treatments. Patients with TRD sometimes have comorbid cervical stiffness. This observational study aims to examine the association of local modulation of cervical muscles with TRD and to learn the involvement of the parasympathetic nervous system in the underlying mechanism.

**Methods:**

A total of 1103 hospitalized patients with TRD who were resistant to outpatient care were enrolled between May 2006 and October 2021. All patients underwent local modulation of the cervical muscles by physical therapy during hospitalization. The presence or absence of TRD and whole-body disorders, such as headache, dazzling, cervical stiffness, and cardiovascular and gastrointestinal disorders, was determined by the patient’s subjectivity using the self-rated medical interview sheet at admission and discharge. Pupil light reflex parameters were also measured at admission and discharge using a binocular infrared pupilometer.

**Results:**

The improvement rate of TRD during hospitalization was 72.1%, and did not differ significantly by sex, age, and hospitalization period. The improvement of TRD showed a strong association with those of cervical stiffness and dazzling, a pupil light reflex disorder (*p* < 0.001: odds ratios = 12.76 and 6.39, respectively), but not with those of headache or cardiovascular and gastrointestinal disorders (*p* > 0.05). In the TRD-improved patients, the pupil light reflex parameters representative of the parasympathetic nervous system function ameliorated: pupil diameter decreased, while constriction rate and velocity increased during hospitalization. In contrast, little amelioration of the parameters was seen in the TRD-unimproved patients.

**Conclusions:**

Cervical muscle stiffness may be associated with TRD, possibly through dysfunction of the parasympathetic nervous system.

**Trial registration:**

ID: UMIN000040590. First registration date: 30/05/2020.

**Supplementary Information:**

The online version contains supplementary material available at 10.1186/s12891-022-05860-2.

## Background

Although major depressive disorder is a representative public health problem, the diagnostic criteria have not yet been defined [1, 2]. Furthermore, despite a wide variety of treatments, including antidepressants, mood stabilizers, exercise, education, and psychotherapy, a significant percentage of patients with this disorder do not respond adequately to them [3, 4]. The patients who fail to respond to two or more treatments are diagnosed with treatment-resistant depression (TRD) [5–7], which increases mortality due to suicides and imposes an enormous burden on society [8–10].

TRD can be accompanied by indefinite disorders in the whole body [8, 11, 12]. In our clinical experience, we have observed a potential trend in indefinite whole-body disorders including TRD that occasionally coincide with cervical stiffness, and proposed a new medical concept called “cervical neuro-muscular syndrome” [13]. Our previous study of 1863 patients showed that about 60% of patients with cervical disorders have comorbid depression [14]. We have attempted local and physical modulation of the cervical stiffness to treat these indefinite whole-body disorders. Among physical therapies, low-frequency electrical stimulation [15, 16] and far-infrared irradiation [17] are reportedly effective at ameliorating the stiffness. The previous study showed that the combined application of these two physical therapies to the cervical muscles significantly decreased the number of indefinite whole-body disorders [14]. Also, our studies disclosed that the physical therapies to the cervical muscles were effective in patients with whiplash-associated disorders [18] and myalgic encephalomyelitis/chronic fatigue syndrome [19].

As the underlying mechanism, we proposed dysfunction of the parasympathetic nervous system which might possibly be caused by the cervical stiffness [14, 19]. The parasympathetic nervous system controls rest, recovery from stress, and maintaining homeostasis, while the sympathetic nervous system, the other autonomic nervous system, plays an excitatory role in a stressful situation. To evaluate parasympathetic nervous system function, the pupil light reflex is known to be a representative indicator; the constrictor muscle of the pupil decreases in diameter under the control of the ciliary ganglion, which is activated and innervated by a preganglionic autonomic nerve fiber [20, 21]. The pupil light reflex has been used to evaluate autonomic nerve dysfunction in patients with Parkinson’s disease, Alzheimer’s disease, and diabetes mellitus [22–24].

This study examined the association of improvement of TRD with those of whole-body disorders using physical therapies [15–17] as a method to ameliorate cervical stiffness. Furthermore, the involvement of the parasympathetic nervous system was investigated by comparing the changes in the pupil light reflex parameters during hospitalization between patients with and without TRD improvement.

## Methods

### Study design

This study is an observational study that compared patients with versus without improvement of TRD.

### Patients

This study was conducted with the approval of the institutional review board of Tokyo Neurological Center and Matsui Hospital. Written informed consent was obtained from all participants or a parent or guardian for participants under 18 years old.

The presence or absence of symptoms was based on the patient’s subjectivity using the self-rated medical interview sheet, a part of which is shown as Supplementary Table 1. Since the diagnostic criteria of TRD have not yet been defined, we have diagnosed patients who reported all four representative symptoms; depressive condition, bedridden condition, lethargy, and uneasy feeling, as the major depressive disorder, based on previous reports [1, 2]. For all participants, the self-rated medical interview sheets documenting the presence or absence of the four representative symptoms were collected at admission and discharge. Also, the interview sheets documenting the presence or absence of representative five whole-body disorders; headache, dazzling, cervical stiffness, cardiovascular disorders, and gastrointestinal disorders, that have been reported to accompany TRD [8, 11, 12] were collected at admission and discharge. Patients who reported one or more symptoms among palpation, chest tightness, thermoregulation disorder and poor circulation were diagnosed with cardiovascular disorders, while those with nausea or stomachache and diarrhea or constipation were diagnosed with gastrointestinal disorders (Supplementary Table 1).

Among patients with major depressive disorder, who visited our institution between May 2006 and October 2021, we enrolled 1277 who were diagnosed with TRD due to failure to respond to two or more outpatient treatments [5–7] and were hospitalized. Outpatient treatments at our institution included antidepressants, mood stabilizers, exercise, education, and psychotherapy, but did not include physical therapy for cervical muscles. This is because the outpatient facility at our institution, like most hospitals in Japan, does not have equipment for the physical therapy, whereas the inpatient facility does.

The TRD patients who recovered from two or more symptoms among the four representative symptoms of the major depressive disorder were defined to be improved (Supplementary Table 1). Similarly, for patients with cardiovascular and gastrointestinal disorders, those who recovered from one or more symptoms were defined to be improved.

Hospitalization and discharge were decided with consent between patients and physicians without any objective criteria. Although our institution has had evidence that physical therapy decreased the number of indefinite whole-body disorders including depression [14], there have been no reports showing the success of physical therapy on TRD nor information of the effects on the patients. Hence, it is improbable that the decision of hospitalization or discharge was dependent on or biased by the patient’s prejudice. Although patients were allowed to stay until they felt improved, those hospitalized for 10–120 days were included in this study. Patients who refused the pupil light reflex test, those who were diagnosed with specific diseases in other organs after admission, or those who discharged themselves from the hospital based on their judgment for unknown reasons, were excluded.

### Intervention

All patients underwent low-frequency electrical stimulation and far-infrared irradiation [15–17] applied to the cervical muscles for 15 minutes two times daily throughout hospitalization (Supplementary Fig. 1A). No other intervention, including medication, injection, cervical traction or fixation, exercise, education, or psychotherapy, was performed under the written informed consent of participants. The approval of this management by our institution was because the patients have already exhausted all possibilities of outpatient treatments. Also, our institution has had evidence that physical therapies without any intervention decreased the number of indefinite whole-body disorders including depression [14], suggesting a possibility of the effect.

Silver spike point (SSP; Nihon Medix, Chiba, Japan) and pain topra (LCF-30; Celcom, Inc., Fukuoka, Japan) were used for the low-frequency electrical stimulation and the far-infrared ray irradiation, respectively. The physical therapies were applied from all directions, at least to one point in the anterior, posterior, right, and left regions, depending on palpation by physicians to determine the muscle lesions [13] (Supplementary Fig. 1B).

Assessment of the pupil light reflex was performed at admission and discharge using a binocular infrared pupilometer (Iriscoder Dual C10641; Hamamatsu Photonics, Shizuoka, Japan) as previously reported [25, 26] (Supplementary Fig. 2). Briefly, a red-light stimulus was presented to the right eye, and three parameters were measured in the right eye: initial pupil diameter (mm) before the stimulus, percentage constriction rate ([pupil diameter before the light stimulus – minimum pupil diameter after the light stimulus]/[pupil diameter before the light stimulus] × 100), and the constriction velocity (mm/second).

### Statistical analysis

Statistical analyses were performed using SPSS 16.0 J for Windows. Two-sided *p*-values less than 0.05 were considered statistically significant. As the sample size (*n* = 1103) was sufficient, the central limit theorem could be applied to confirm that the data were normally distributed and that violating the normality assumption would not cause major problems [27]. Hence, the paired Student’s t-test was used to examine the difference in the number of disorders at admission versus discharge, while the unpaired t-test was used to compare means between the improved and unimproved TRD groups. In the univariate and multivariate logistic regression analysis, all variables were force entered into the multivariate model. Forward stepwise multivariate logistic regression analysis was also performed. The best model was selected based on the likelihood ratio test results.

## Results

### Participant flow and backgrounds

**Fig. 1:**
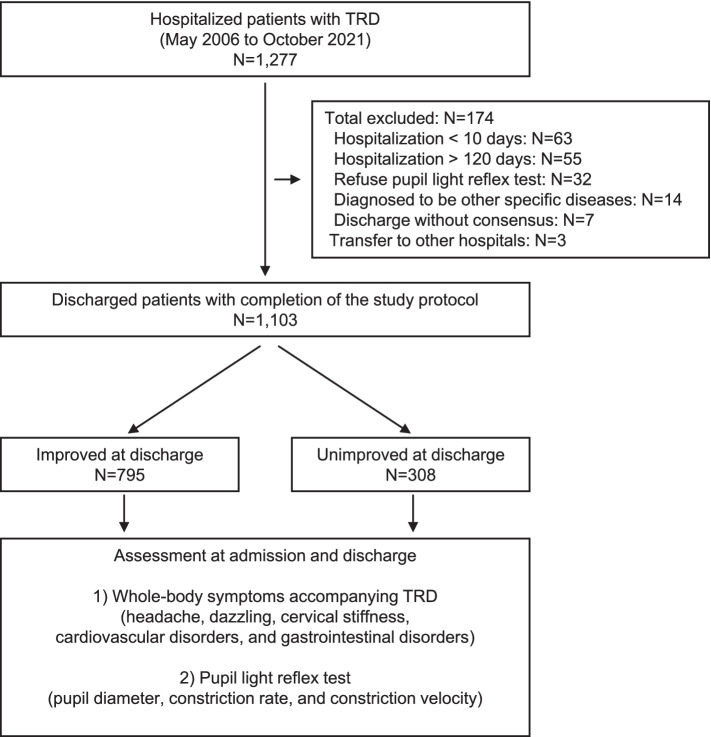
Flow chart of participant enrollment and study design

Figure 1 shows a flowchart of the patient enrollment process of the present study. A total of 1277 hospitalized patients diagnosed with TRD [5–7] were initially enrolled in this study. Of them, 174 were excluded after enrollment, including 63 who were discharged from the hospital after less than ten days, 55 who were hospitalized for more than 120 days, 32 who refused pupil light reflex test, 14 who were diagnosed with specific diseases in other organs after admission, seven who discharged themselves from the hospital based on their judgment with unknown reasons, and three who were transferred to other hospitals for treatment of specific diseases. After the removal of these patients, 1103 completed the study protocol. Among them, 795 (72.1%) were diagnosed as having improved from TRD at discharge, while 308 (27.9%) remained unimproved.

The presence or absence of whole-body disorders and the pupil light reflex parameters at admission and discharge were compared between the TRD-improved and -unimproved patients. Table 1 shows the baseline characteristics of the 1103 participants (383 men, 720 women) with a mean age of 49.5 ± 18.1 years (mean ± standard deviation) and a mean hospitalization period of 66.9 ± 25.4 days. The improvement rate was not significantly altered by sex (men versus women), age strata (< 50 versus ≥50 years), or hospitalization period (10–60 versus 61–120 days) (*p* > 0.05).

**Table 1:** Baseline characteristics of the study participants with versus without TRD recovery

		Number (%)	Number (%)	Number (%)	
		Total	Improved	Unimproved	*P*-value
Variables		1103 (100.0)	795 (72.1)	308 (27.9)	
Sex	Men	383 (34.7)	287 (74.9)	96 (25.1)	0.213
	Women	720 (65.3)	508 (70.6)	212 (29.4)	
Age strata (years old)	< 50	632 (57.3)	470 (74.4)	162 (25.6)	0.152
	≥50	471 (42.7)	325 (69.0)	146 (31.0)	
Hospitalization period (days)	10–60	434 (39.3)	306 (70.5)	128 (29.5)	0.631
	61–120	669 (60.7)	486 (72.6)	183 (27.4)	

### Relationship between TRD improvement and related disorders

Among the representative five disorders accompanying TRD [8, 11, 12], more than 75% of TRD patients reported headache and cervical stiffness at admission, while 45.4, 23.2, and 59.3% reported dazzling, cardiovascular disorders, and gastrointestinal disorders, respectively (Table 2). On the other hand, the improvement rates of these disorders in the total population were more than 70% in patients with dazzling and cervical stiffness, but less than 70% in those with headache, cardiovascular and gastrointestinal disorders (Table 2). This was reproducible in the TRD-improved population, suggesting a positive association of improvements of dazzling and cervical stiffness with that of TRD.

**Table 2:** Number (percentage) of patients with representative five disorders accompanying TRD at admission and those with and without improvement at discharge

	Total (*n* = 1103)	TRD improved (*n* = 795; 72.1%)	TRD unimproved (*n* = 308; 27.9%)
Headache at admission	862 (78.2)	618 (71.7)	244 (28.3)
Improved at discharge	451 (52.3)	324 (52.4)	127 (52.0)
Unimproved at discharge	411 (47.7)	294 (47.6)	117 (48.0)
Dazzling at admission	501 (45.4)	358 (71.5)	143 (28.5)
Improved at discharge	378 (75.4)	281 (78.5)	97 (67.8)
Unimproved at discharge	123 (24.6)	77 (21.5)	46 (32.2)
Cervical stiffness at admission	839 (76.1)	683 (81.4)	156 (18.6)
Improved at discharge	591 (70.4)	580 (84.9)	11 (7.1)
Unimproved at discharge	248 (29.6)	103 (15.1)	145 (92.9)
Cardiovascular disorders at admission	256 (23.2)	195 (76.2)	61 (23.8)
Improved at discharge	162 (63.3)	125 (64.1)	37 (60.7)
Unimproved at discharge	94 (36.7)	70 (35.9)	24 (39.3)
Gastrointestinal disorders at admission	654 (59.3)	340 (52.0)	314 (48.0)
Improved at discharge	423 (64.7)	187 (55.0)	236 (75.2)
Unimproved at discharge	231 (35.3)	153 (45.0)	78 (24.8)

The logistic regression analysis confirmed that the improvement (versus non-improvement) of dazzling and cervical stiffness showed a strong association with that of TRD (*p* < 0.001; odds ratios = 6.39 and 12.76, respectively), while headache and cardiovascular and gastrointestinal disorders did not (*p* > 0.05) (Table 3). These results demonstrate that TRD improvement during hospitalization is associated with the pupil light reflex which is impaired in the dazzling, as well as with cervical stiffness.

**Table 3:** Odds ratio (95% CI) of the improvement (vs. non-improvement) of indefinite whole-body disorders to that of TRD by logistic regression analysis

	Number (%) of patients	Number (%) of improved patients	Odds ratio	95% CI	*P*-value
Headache	862 (78.2)	451 (52.3)	2.60	0.87–4.51	> 0.05
Dazzling	501 (45.4)	378 (75.4)	6.39	3.45–11.92	< 0.001
Cervical stiffness	839 (76.1)	591 (70.4)	12.76	8.05–21.42	< 0.001
Cardiovascular disorders	256 (23.2)	162 (63.3)	4.65	0.71–8.87	> 0.05
Gastrointestinal disorders	654 (59.3)	423 (64.7)	3.79	0.93–6.90	> 0.05

*CI* Confidence of interval

### Pupil light reflex test

**Fig. 2:**
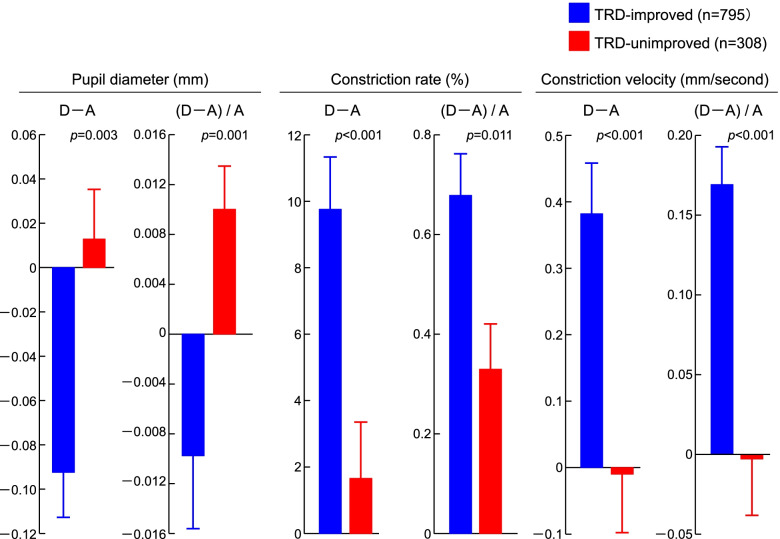
Changes (D–A) in three parameters of the pupil light reflex test (pupil diameter, constriction rate, and constriction velocity) between admission and discharge, as well as the ratio adjusted by those at admission ([D–A]/A). Blue and red bars show the mean ± standard error of treatment-resistant depression (TRD)-improved patients and -unimproved patients, respectively, with the *p*-values of differences between them

We further examined the possible involvement of parasympathetic nervous system function by comparing three pupil light reflex parameters between admission and discharge. We first confirmed that the changes (D–A) of pupil reflex parameters (pupil diameter, constriction rate, and constriction velocity) were not significantly different between shorter (10–60 days) and longer (61–120 days) hospitalization periods, just like the improvement rate of cervical stiffness (Supplementary Table 2). In the TRD-improved patients, both pupil diameter (D–A = –0.091 ± 0.021 mm; mean ± standard error) and the ratio adjusted by those at admission ([D–A]/A = –0.009 ± 0.006) decreased during hospitalization, while constriction rate (D–A = 9.82 ± 1.64%) and the ratio ([D–A]/A = 0.66 ± 0.09), as well as constriction velocity (D–A = 0.38 ± 0.08 mm/second) and the ratio ([D–A]/A = 0.162 ± 0.033) were increased (Fig. 2). These suggest that the physical therapy ameliorated pupil light reflex representing parasympathetic nervous system function during hospitalization. In the TRD-unimproved patients, however, amelioration of the pupil light reflex parameters; decrease in pupil diameter and increases in constriction rate and velocity, were not seen or were significantly lower than in the TRD-improved patients (*p* = 0.003, *p* < 0.001, and *p* < 0.001 in D–A; *p* = 0.001, *p* = 0.011, and *p* < 0.001 in [D–A]/A, respectively). Taken together, the improvement of TRD was positively associated with that of pupil light reflex and parasympathetic nervous system function.

## Discussion

This study showed a strong association of the improvement of TRD with those of cervical stiffness and dazzling which is the disorder of pupil light reflex and possibly that of parasympathetic nervous system function. We, therefore, propose that cervical stiffness may be associated with TRD, possibly through dysfunction of the parasympathetic nervous system.

The canonical pathway that regulates the pupil light reflex is such that the ganglion cell axons project to the Edinger-Westphal nucleus in the midbrain, where the preganglionic parasympathetic neuron fibers in the oculomotor nerve which are activated and command the constrictor muscles of the pupil [20, 21]. Although the oculomotor nerve does not run through the cervical region, another non-canonical pathway via the afferent parasympathetic neuron fiber in the vagus nerve arising from the brainstem and running alongside cervical muscles down to the thoracic and abdominal viscera [28] may be involved in the regulation of the pupil light reflex. In fact, the cervical vagus nerve stimulation (VNS) therapy was approved by the U.S. Food and Drug Administration as a treatment for TRD with the advantages of sustained therapeutic response and favorable safety profile [29–32]. Considering that the cervical VNS is supposed to work through the parasympathetic nervous system, the present results may not be much novel or surprising. However, the advantage of the present physical therapy is that this is less invasive than VNS which needs a surgical procedure.

The causal relationship between improvements in cervical stiffness and the parasympathetic nervous system remains unclarified. Our analysis of the changes (D–A and [D–A]/A) of the pupil light reflex parameters showed that pupil diameter decreased while constriction rate and velocity increased in the cervical stiffness-improved patients (Supplementary Fig. 3). In the cervical stiffness-unimproved patients, amelioration of the pupil light reflex parameters was lower than that in the cervical stiffness-improved patients. These suggest that improvement of cervical stiffness was positively correlated with the amelioration of pupil light reflex and parasympathetic nervous system. However, the differences of the parameters between stiffness-improved and -unimproved patients, as shown by the *p*-values, were smaller than those between TRD-improved patients and -unimproved patients (Fig. 2). Hence, there might be other mechanisms underlying the improvements of parasympathetic nervous system and TRD, independent of that of the cervical stiffness. In fact, both electrical stimulation and far-infrared irradiation are reported to cause nerve regeneration and repair directly [33, 34]. Furthermore, the vagus nerve does not travel inside the cervical muscles, but runs along the carotid sheath in the neck. The present physical therapies were applied from all directions; anterior, posterior, and lateral to the cervical region (Supplementary Fig. 1B). Further studies on the relationship between the effect and the application site associated with the vagus nerve localization would possibly clarify whether the effect is direct to the vagus nerve or indirect through the cervical muscle modulation.

Most patients with TRD have substantial mental comorbidity and disorders related to muscle tension. Although there was a strong association of improvements between cervical stiffness and TRD (Table 3), a crucial limitation of this study is that it remains unclarified whether cervical stiffness is the cause or effect of TRD. In addition, the presence or absence of cervical stiffness was determined by the patient’s subjectivity using the self-rated medical interview sheet. More quantitative and objective measurements, e.g., ultrasound elastography technique used in oncology and now applied to measure the mechanical properties of the muscle in patients with multiple sclerosis spasticity [35], may be needed. A prospective study using shear-wave elastography [36], a representative ultrasound elastography technique, to measure cervical muscle stiffness is now underway.

Although about 80% of the TRD patients complained of headache at admission, the improvement of TRD was not significantly associated with that of headache (Tables 2 and 3), suggesting a different mechanism between TRD and headache underlying the effects of physical therapy. In addition to the upper cervical dysfunction of facet joints or occipital muscles, we speculate that the headache in this study may at least partly be the cervicogenic type whose diagnostic criteria are unilateral headache without side-shift and pain starting in the neck and spreading to the fronto-ocular area [37]. Since it is reported that upper cervical spine mobilization demonstrates more clinical benefits than massage therapy of the cervical muscles for this type of headache [38], it is expected that the present physical therapy for modulation of the cervical muscles is not effective for this headache.

This study is the first to show that physical therapy delivered to the cervical muscles improved more than 70% of the patients with TRD. However, we would like to emphasize that the present study is an observational study that examined the association of local modulation of cervical muscles with TRD, but not a prospective study aiming at the clinical application of specific physical therapies. In other words, we used physical therapies as a method for the local modulation of cervical muscles. Although the patients in this study did not receive any other interventions during the hospitalization, we cannot deny the possibility that TRD might improve through long-term rest or changing air. As a trial study aiming at the clinical application of the physical therapies to TRD treatment, a control group of patients who did not undergo the therapies to cervical muscles during hospitalization would be essential. However, physical therapy performed two times daily for a mean of 66.9 days per patient is too costly for health care providers and patients. Since we believe that local modulation of the cervical muscles by pharmacological intervention would also effectively treat TRD, we are now planning a clinical trial that examines the effects of more feasible treatments like a topical muscle-relaxant poultice or ointment in patients with TRD.

## Conclusions

Cervical muscle stiffness may be associated with TRD, possibly through dysfunction of the parasympathetic nervous system, although the causality requires further studies.

## Supplementary Information

**Additional file 1.**

**Additional file 2.**

## Data Availability

The datasets used and analyzed during the current study are available from the corresponding author upon reasonable request.

## Abbreviations

TRD: Treatment-resistant depression
SSP: Silver spike point
VNS: Vagus nerve stimulation
