# Supplementary material for: Cervical muscle stiffness and parasympathetic nervous system improvements for treatment-resistant depression
Source: BMC Musculoskelet Disord. 2022 Oct 11;23:907. doi: 10.1186/s12891-022-05860-2 (PMC9552456; doi:10.1186/s12891-022-05860-2)
Supplement: Supplementary file 1 — Additional file 1. [file 12891_2022_5860_MOESM1_ESM.docx]

**Supplementary Table 1.** A part of the self-rated medical interview sheet documenting symptoms of major depressive disorder and five whole-body disorders

| Major depressive disorder |
| --- |
| Depressive condition  Bedridden condition  Lethargy  Uneasy feeling |
| Headache |
| Cervical stiffness |
| Dazzling |
| Cardiovascular disorders |
| Palpation  Chest tightness  Thermoregulation disorder  Poor circulation |
| Gastrointestinal disorders |
| Nausea or stomachache  Diarrhea or constipation |

**Supplementary Table 2.** Comparison of improvement rate of cervical stiffness and changes of pupil light reflex parameters between patients with shorter (10-60 days) and longer (61-120 days) hospitalization periods

| Hospitalization period (days) | Improvement rate of cervical stiffness  (%) | Changes of pupil light reflex parameters (D－A) | | |
| --- | --- | --- | --- | --- |
|  |  | Pupil diameter (mm) | Constriction rate  (%) | Constriction velocity  (mm/second) |
| 10-60 | 70.8 ± 10.1 | –0.065 ± 0.019 | 7.83 ± 0.14 | 0.28 ± 0.09 |
| 61-120 | 70.1 ± 8.3 | –0.063 ± 0.030 | 7.46 ± 0.37 | 0.27 ± 0.21 |
| P-value | >0.05 | >0.05 | >0.05 | >0.05 |

mean ± standard error
