# Supplementary material for: Cervical muscle stiffness and parasympathetic nervous system improvements for treatment-resistant depression
Source: BMC Musculoskelet Disord. 2022 Oct 11;23:907. doi: 10.1186/s12891-022-05860-2 (PMC9552456; doi:10.1186/s12891-022-05860-2)
Supplement: Supplementary file 2 — Additional file 2. [file 12891_2022_5860_MOESM2_ESM.pdf]

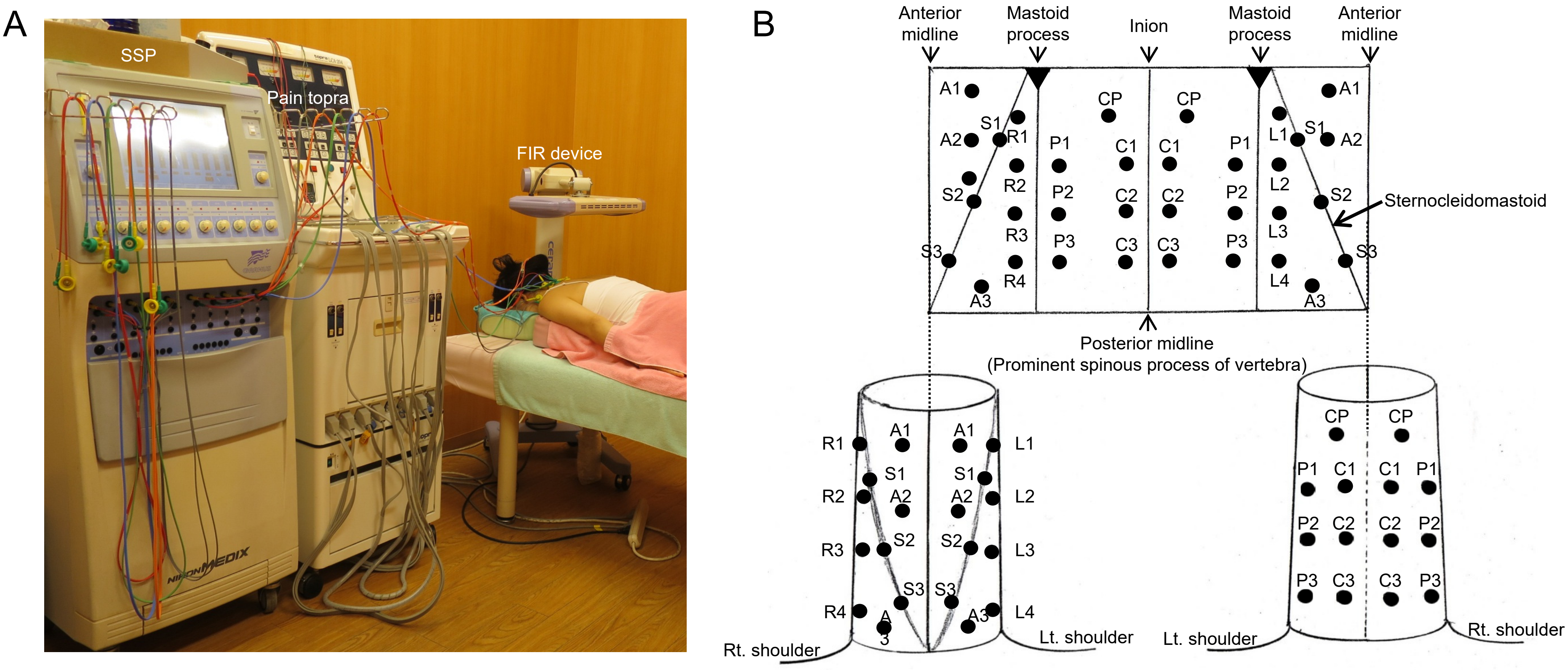

Supplementary Figure 1. (**A**) Combined application of two physical therapies to the cervical muscles using low-frequency electrical stimulation by silver spike point (SSP) and far-infrared irradiation (FIR) by pain topra. (**B**) Points of the neck where the physical therapies were applied. The physical therapies were applied from all directions; at least to one point at anterior (A or S), posterior (C, P, or CP), right (R), and left (L) regions, depending on palpation by physicians to determine the muscle lesions.

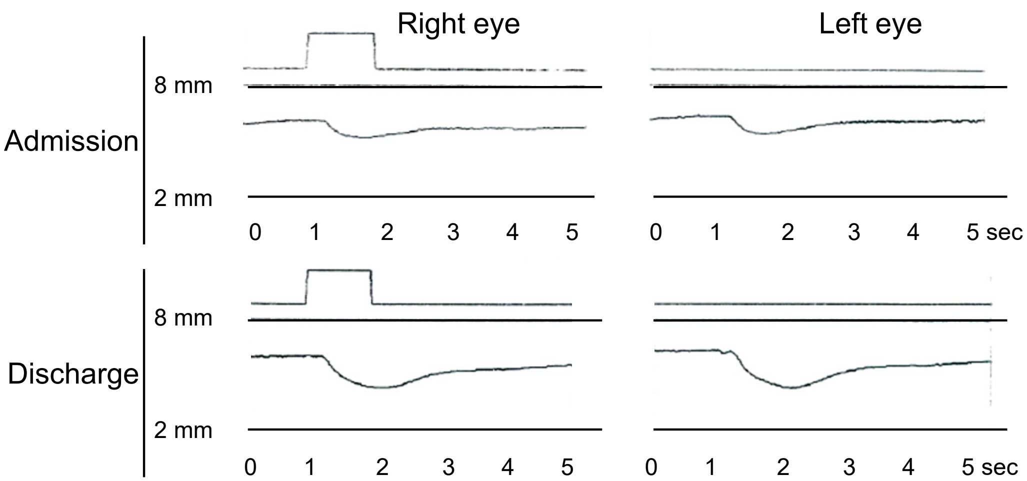

Pupil light reflex test
(Admission → Discharge)

Pupil diameter (mm): 6.3 → 6.4
Constriction rate (%): 8.0 → 21.0
Constriction velocity (mm/second): 3.1 → 4.3

Supplementary Figure 2. An example of a pupil light reflex test using the binocular infrared pupilometer (Iriscoder).
The top lines show light stimulus of the right eye only. The curved lines below show the pupil diameter (mm). Three parameters on the pupil diameter curves were measured in the right eye: pupil diameter (mm) before the light stimulus; constriction rate (%), (pupil diameter before the light stimulus − minimum pupil diameter after the light stimulus)/(pupil diameter before the light stimulus) ×100; and constriction velocity (mm/second).

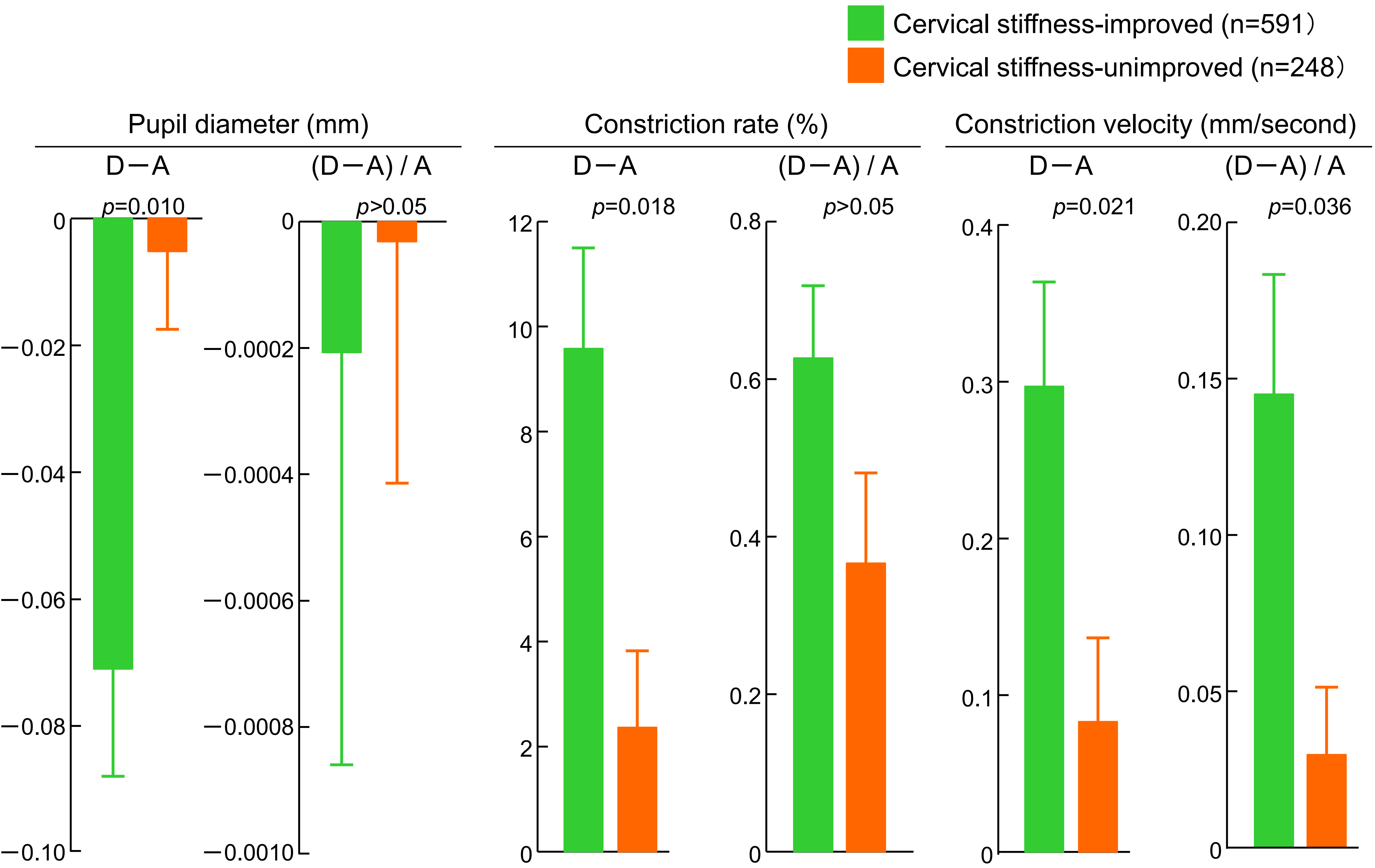

Supplementary Figure 3. Changes (D–A) in the pupil light reflex parameters (pupil diameter, constriction rate, and constriction velocity) between admission and discharge, as well as the ratio adjusted by those at admission ([D–A]/A). Green and orange bars show the mean ± standard error of cervical stiffness-improved patients and -unimproved patients, respectively, with the *p*-values of differences between them.
